# On maximum likelihood estimation of the concentration parameter of von Mises–Fisher distributions

**DOI:** 10.1007/s00180-013-0471-0

**Published:** 2013-12-13

**Authors:** Kurt Hornik, Bettina Grün

**Affiliations:** 1 Institute for Statistics and Mathematics, WU Wirtschaftsuniversität Wien, Vienna, Austria; 2 Institut für Angewandte Statistik, Johannes Kepler Universität Linz, Linz, Austria

**Keywords:** Maximum likelihood, Modified Bessel function ratio, Numerical approximation, von Mises–Fisher distribution

## Abstract

Maximum likelihood estimation of the concentration parameter of von Mises–Fisher distributions involves inverting the ratio $R_\nu = I_{\nu +1} / I_\nu$ of modified Bessel functions and computational methods are required to invert these functions using approximative or iterative algorithms. In this paper we use Amos-type bounds for $R_\nu$ to deduce sharper bounds for the inverse function, determine the approximation error of these bounds, and use these to propose a new approximation for which the error tends to zero when the inverse of $R_\nu$ is evaluated at values tending to $1$ (from the left). We show that previously introduced rational bounds for $R_\nu$ which are invertible using quadratic equations cannot be used to improve these bounds.

## Introduction

A random unit length vector in $\mathbb {R}^d$ has a *von Mises–Fisher* (or Langevin) distribution with parameter $\theta \in \mathbb {R}^d$ if its density with respect to the uniform distribution on the unit hypersphere $\mathbb {S}^{d-1} = \{x \in \mathbb {R}^d: \Vert x\Vert = 1 \}$ is given by

$$\begin{aligned} f(x|\theta ) = e^{\theta ' x} / {}_{0}F_{1}(; d/2; \Vert \theta \Vert ^2 / 4), \end{aligned}$$

where

$$\begin{aligned} {}_{0}F_{1}(; \nu ; z) = \sum _{n=0}^\infty \frac{\Gamma (\nu )}{\Gamma (\nu +n)}\frac{z^n}{n!} \end{aligned}$$

is a generalized hypergeometric series and related to the modified Bessel function of the first kind $I_\nu$ via

$$\begin{aligned} {}_{0}F_{1}(; \nu + 1; \kappa ^2 / 4) = \frac{I_\nu (\kappa ) \Gamma (\nu + 1)}{(\kappa / 2)^\nu } \end{aligned}$$

(e.g., Mardia and Jupp 1999, p. 168).

We note that the von Mises–Fisher distribution is commonly parametrized as $\theta = \kappa \mu$, where $\kappa = \Vert \theta \Vert$ and $\mu \in \mathbb {S}^{d-1}$ are the *concentration* and *mean direction* parameters, respectively (if $\theta \ne 0$, $\mu$ is uniquely determined as $\theta / \Vert \theta \Vert$).

Using the common parametrization by $\kappa$ and $\mu$, the log-likelihood of a sample $x_1, \ldots , x_n$ from the von Mises–Fisher distribution is given by

$$\begin{aligned} - n \log ({}_{0}F_{1}(; d/2; \kappa ^2/4)) + \kappa \mu ' r, \end{aligned}$$

where $r = \sum _{i=1}^n x_i$ is the resultant vector (sum) of the $x_i$. Using recursions for the modified Bessel functions (e.g., Watson 1995, p. 71), one can show that the negative log-derivative of $\kappa \mapsto \log ({}_{0}F_{1}(; d/2; \kappa ^2/4))$ equals $A_d(\kappa ) = R_{d/2-1}(\kappa )$, where $R_\nu (t) = I_{\nu +1}(t) / I_{\nu }(t)$. The maximum likelihood estimators are thus obtained by taking $\hat{\mu } = r / \Vert r\Vert$ and solving

$$\begin{aligned} R_{d/2-1}(\hat{\kappa }) = \rho , \end{aligned}$$

where $\rho = \Vert r\Vert / n$ is the mean resultant length (Schou 1978).

It can be shown (e.g., Schou 1978) that for $\nu \ge 0$, $R_\nu$ is strictly increasing, and satisfies the Riccati equation $R_\nu '(t) = 1 - ((2\nu + 1) / t) R_\nu (t) - R_{\nu }(t)^2$. As $R_\nu$ and hence also its derivatives can efficiently be computed via its Perron or Gauss continued fraction representation (Gautschi and Slavik 1978; Tretter and Walster 1980; Song et al. 2012), solving $R_{\nu }(t) = \rho$ should conveniently be achievable by standard iterative root finding techniques, provided that good starting approximations are available (which is particularly important in the right tail of $R_\nu$ where $R_\nu$ is rather “flat”).

Dhillon and Sra (2003) and subsequently Banerjee et al. (2005) suggest the approximation

1 $$\begin{aligned} R_{\nu }^{-1}(\rho ) \approx \frac{\rho }{1 - \rho ^2} (2(\nu +1) - \rho ^2) =: Q_\nu (\rho ) \end{aligned}$$

obtained by truncating the Gauss continued fraction representation of $R_\nu$ and adding a correction term “determined empirically”, pointing out that this initial approximation can subsequently be improved by Newton–Raphson iterations. Sra (2012) suggests to use exactly two such iterations.

Tanabe et al. (2007) show that for $\nu \ge 0$ and $0 \le \rho < 1$,

$$\begin{aligned} R_{\nu }^{-1}(\rho ) = \frac{\rho }{1 - \rho ^2} (2(\nu +1) - c) \end{aligned}$$

for some suitable $0 \le c = c(\nu , \rho ) \le 2$, or equivalently,

2 $$\begin{aligned} \frac{2\nu \rho }{1 - \rho ^2} \le R_{\nu }^{-1}(\rho ) \le \frac{2(\nu +1)\rho }{1 - \rho ^2}, \end{aligned}$$

with the Dhillon–Sra approximation assuming $c \approx \rho ^2$. The upper and lower bound differ by $2\rho / (1 - \rho ^2)$ which is independent of $\nu$ but tends to infinity as $\rho \rightarrow 1-$. Tanabe et al. (2007) also suggest to use the “mid-point” approximation with $c = 1$, i.e., $R_\nu ^{-1}(\rho ) \approx (2\nu + 1) \rho / (1 - \rho ^2)$ as the starting value for iterative schemes for solving $R_\nu (t) = \rho$, such as the fixed-point iteration $t_{n+1} = t_n \rho / R_\nu (t_n)$.

In this paper, we use a family of bounds for $R_\nu$ first introduced in Amos (1974) to provide substantially sharper bounds for $R_\nu ^{-1}$, which have approximation error at most $3\rho /2$, and use these results to suggest a new approximation. We establish that these improved bounds also hold for the Dhillon–Sra approximation, which thus has the same maximal approximation error. We also show that the error of the suggested new approximation tends to zero for $\rho \rightarrow 1-$, whereas the error tends to $-1/2$ for the Dhillon–Sra approximation, which thus is too large for large $\rho$. Finally, we investigate whether the rational bounds for $R_\nu$ developed by Nåsell (1978) can be used to obtain improved explicit bounds for $R_{\nu }^{-1}$, and show that for the rational bounds which can be inverted by solving quadratic equations, no such improvement is possible.

## Amos-type bounds

Let

$$\begin{aligned} G_{\alpha ,\beta }(t) = \frac{t}{\alpha + \sqrt{t^2 + \beta ^2}}, \end{aligned}$$

where in what follows we take $\beta \ge 0$ without loss of generality. Amos (1974) gives the bounds

$$\begin{aligned} G_{\nu +1/2,\nu +3/2}(\kappa ) \le R_\nu (\kappa ) \le G_{\nu +1/2,\nu +1/2}(\kappa ), \qquad \kappa , \nu \ge 0 \end{aligned}$$

(Equation 16) and

$$\begin{aligned} G_{\nu +1,\nu +1}(\kappa ) \le R_\nu (\kappa ) \le G_{\nu ,\nu +2}(\kappa ) \le G_{\nu ,\nu }(\kappa ), \qquad \kappa , \nu \ge 0 \end{aligned}$$

(Equations 9 and 11). The bounds are actually valid for larger $\nu$ domains (see for example Nåsell 1978; Yuan and Kalbfleisch 2000). It is trivial that $G_{\nu +1/2,\nu +1/2}(t) < G_{\nu ,\nu }(t)$ for all $t > 0$. For $\Delta (t) = (\nu +1/2) + \sqrt{t^2 + (\nu +3/2)^2} - ((\nu +1) + \sqrt{t^2 + (\nu +1)^2})$ we have $\Delta (0) = 0$ and $\Delta '(t) = t / \sqrt{t^2 + (\nu +3/2)^2} - t / \sqrt{t^2 + (\nu +1)^2}$, which is negative for all $t > 0$. Thus, $\Delta (t) < 0$ for all $t > 0$ and hence $G_{\nu +1/2,\nu +3/2}(t) > G_{\nu +1,\nu +1}(t)$ for all $t > 0$.

Let

$$\begin{aligned} \beta _{SS}(\nu ) = \sqrt{(\nu + 1/2)(\nu + 3/2)}. \end{aligned}$$

Simpson and Spector (1984) show that with $v_\nu (t) = t / R_{\nu }(t)$, $v_\nu (t)^2 - (2\nu + 1) v_\nu (t) - (t^2 + \nu + 1/2) > 0$ for all $\nu \ge 0$ and $t > 0$, which is readily seen to imply $v_{\nu }(t) \ge \nu +1/2 + \sqrt{t^2 + \beta _{SS}(\nu )^2}$ and hence $R_\nu (t) \le G_{\nu +1/2,\beta _{SS}(\nu )}(t)$, which is clearly smaller than $G_{\nu +1/2,\nu +1/2}(t)$ for all $t > 0$.

Altogether, we thus have that for $\nu \ge 0$ and $t \ge 0$,

3 $$\begin{aligned} G_{\nu +1/2,\nu +3/2}(t) \le R_{\nu }(t) \le \min \left( G_{\nu ,\nu +2}(t), G_{\nu +1/2,\beta _{SS}(\nu )}(t) \right) . \end{aligned}$$

What makes these Amos-type bounds particularly attractive is that they can be inverted explicitly, as shown in the following lemma.

### **Lemma 1**

Let $\alpha \ge 0$ and $\alpha + \beta > 0$. Then $G_{\alpha ,\beta }$ is strictly increasing on $[0, \infty )$, and for all $0 \le \rho < 1$ the equation $G_{\alpha ,\beta }(t) = \rho$ has a unique solution $t = G_{\alpha ,\beta }^{-1}(\rho )$ given by

4 $$\begin{aligned} G_{\alpha ,\beta }^{-1}(\rho ) = \frac{\rho }{1 - \rho ^2} \left( \alpha + \sqrt{\rho ^2 \alpha ^2 + (1 - \rho ^2) \beta ^2} \right) . \end{aligned}$$

### *Proof*

The derivative of $G_{\alpha ,\beta }$ is given by

$$\begin{aligned} G_{\alpha ,\beta }'(t)&= \frac{1}{\alpha + \sqrt{t^2 + \beta ^2}} - \frac{t}{(\alpha + \sqrt{t^2 + \beta ^2})^2} \frac{2t}{2\sqrt{t^2 + \beta ^2}} \\&= \frac{\alpha \sqrt{t^2 + \beta ^2} + \beta ^2}{ (\alpha + \sqrt{t^2 + \beta ^2})^2 \sqrt{t^2 + \beta ^2}}, \end{aligned}$$

where the numerator has value $\beta (\alpha + \beta )$ at $t = 0$ and hence is positive for all $t > 0$ if and only if $\alpha \ge 0$ and $\alpha + \beta > 0$ in which case $G_{\alpha ,\beta }$ is strictly increasing, and as $G_{\alpha ,\beta }(0) = 0$ and $\lim _{t \rightarrow \infty } G_{\alpha ,\beta }(t) = 1$ the equation $G_{\alpha ,\beta }(t) = \rho$ has a unique solution for $0 \le \rho < 1$. Now $G_{\alpha ,\beta }(t) = \rho$ iff $t = \rho (\alpha + \sqrt{t^2 + \beta ^2})$, giving $t$ as the larger root of the quadratic equation $(1 - \rho ^2) t^2 - 2 \rho \alpha t + \rho ^2 (\alpha ^2 - \beta ^2) = 0$, so that

$$\begin{aligned} G_{\alpha ,\beta }^{-1}(\rho )&= \frac{1}{2(1-\rho ^2)} \left( 2 \rho \alpha + \sqrt{ 4 \rho ^2 \alpha ^2 - 4 (1 - \rho ^2) \rho ^2 (\alpha ^2 - \beta ^2)} \right) \\&= \frac{\rho }{1 - \rho ^2} \left( \alpha + \sqrt{\rho ^2 \alpha ^2 + (1 - \rho ^2) \beta ^2} \right) \end{aligned}$$

(the smaller root does not converge to $\infty$ as $\rho \rightarrow 1-$).

### **Theorem 1**

Let $\nu \ge 0$ and $0 \le \rho < 1$. Then

5 $$\begin{aligned} \max \left( G_{\nu ,\nu +2}^{-1}(\rho ), G_{\nu +1/2, \beta _{SS}(\nu )}^{-1}(\rho ) \right) \le R_\nu ^{-1}(\rho ) \le G_{\nu +1/2,\nu +3/2}^{-1}(\rho ). \end{aligned}$$

### *Proof*

This follows immediately from combining the previous lemma with Eq. 3.

The above result substantially improves the results of Tanabe et al. (2007). Using Eq. 4, $G_{\alpha ,\alpha }^{-1}(\rho ) = 2\alpha \rho / (1 - \rho ^2)$, so that the lower and upper bound in Eq. 2 equal $G_{\nu ,\nu }^{-1}(\rho )$ and $G_{\nu +1,\nu +1}^{-1}(\rho )$, respectively, and hence correspond to $G_{\nu +1,\nu +1}(t) \le R_\nu (t) \le G_{\nu ,\nu }(t)$, which was already shown to be strictly weaker than the bounds in Eq. 3. We also see that the “mid-point approximation” $R_\nu ^{-1}(\rho ) \approx (2\nu +1) \rho / (1 - \rho ^2)$ equals $G_{\nu +1/2,\nu +1/2}^{-1}(\rho )$, which for positive $\rho$ is strictly smaller than $G_{\nu +1/2,\beta _{SS}(\nu )}^{-1}(\rho )$, and hence strictly under-estimates $R_\nu ^{-1}(\rho )$.

Let

$$\begin{aligned} g(\nu ) = \frac{(\nu + 3/2)}{2 \nu + 1}. \end{aligned}$$

Then $g$ is monotonically decreasing on $[0,\infty )$ with $g(0) = 3/2$ and $\lim _{\nu \rightarrow \infty } g(\nu ) = 1/2$.

### **Theorem 2**

Let $\nu \ge 0$ and $0 \le \rho < 1$. Then

$$\begin{aligned} 0 \le G_{\nu +1/2,\nu +3/2}^{-1}(\rho ) - G_{\nu +1/2,\beta _{SS}(\nu )}^{-1}(\rho ) \le \rho g(\nu ) \end{aligned}$$

and for $$\beta _{SS}(\nu ) \le \beta \le \nu + 3/2$$,

$$\begin{aligned} \big | R_{\nu }^{-1}(\rho ) - G_{\nu +1/2,\beta }^{-1}(\rho ) \big | \le \rho g(\nu ). \end{aligned}$$

### *Proof*

Using the mean value theorem, for $u_1 \ge u_0 >0$ and $\alpha \ge 0$ with a suitable $\tilde{u} \in (u_0, u_1)$,

$$\begin{aligned} 0 \le \left. \sqrt{\alpha + u} \right| _{u=u_0}^{u=u_1} = \frac{u_1 - u_0}{2 \sqrt{\alpha + \tilde{u}}} \le \frac{u_1 - u_0}{2 \sqrt{\alpha + u_0}} \end{aligned}$$

and hence

$$\begin{aligned} 0&\le G_{\nu +1/2,\nu +3/2}^{-1}(\rho ) - G_{\nu +1/2,\beta _{SS}(\nu )}^{-1}(\rho )\\&= \frac{\rho }{1 - \rho ^2} \left. \sqrt{(\nu +1/2)^2 \rho ^2 + \beta ^2 (1 - \rho ^2)} \right| _{\beta =\beta _{SS}(\nu )}^{\beta =\nu +3/2} \\&\le \frac{\rho }{1-\rho ^2} \frac{ (1 - \rho ^2) ((\nu + 3/2)^2 - (\nu +1/2)(\nu +3/2))}{ 2 \sqrt{ (\nu +1/2)^2 \rho ^2 + \tilde{\beta }^2 (1 - \rho ^2) }} \\&\le \frac{\rho (\nu + 3/2)}{2 (\nu + 1/2)}. \end{aligned}$$

For $\beta _{SS}(\nu ) \le \beta \le \nu + 3/2$, both $R_{\nu }^{-1}$ and $G_{\nu +1/2,\beta }^{-1}$ are bounded below by $G_{\nu +1/2,\beta _{SS}(\nu )}^{-1}$ and above by $G_{\nu +1/2,\nu +3/2}^{-1}$, implying that $| R_{\nu }^{-1} - G_{\nu +1/2,\beta }^{-1} | \le G_{\nu +1/2,\nu +3/2}^{-1} - G_{\nu +1/2,\beta _{SS}(\nu )}^{-1}$, whence the result from the bounds on this difference.

### **Corollary 1**

Let $\nu \ge 0$ and $0 \le \rho < 1$. Then

$$\begin{aligned} 0 \le R_{\nu }^{-1}(\rho ) - \max \left( G_{\nu ,\nu +2}^{-1}(\rho ), G_{\nu +1/2, \beta _{SS}(\nu )}^{-1}(\rho ) \right) \le \rho g(\nu ). \end{aligned}$$

### *Proof*

Immediate from Theorems 1 and 2.

### **Theorem 3**

Let $\nu \ge 0$ and $0 \le \rho < 1$. Then

$$\begin{aligned} \max \left( G_{\nu ,\nu +2}^{-1}(\rho ), G_{\nu +1/2, \beta _{SS}(\nu )}^{-1}(\rho ) \right) \le Q_\nu (\rho ) \le G_{\nu +1/2,\nu +3/2}^{-1}(\rho ). \end{aligned}$$

### *Proof*

For $$\beta _\alpha (\rho ) = \sqrt{(\alpha + 1)^2 - \rho ^2}$$,

$$\begin{aligned} \alpha ^2 \rho ^2 + \beta _\alpha (\rho )^2 (1 - \rho ^2)&= \alpha ^2 \rho ^2 + ((\alpha + 1)^2 - \rho ^2) (1 - \rho ^2) = (\alpha + 1 - \rho ^2)^2. \end{aligned}$$

Hence, $\alpha + \sqrt{\alpha ^2 \rho ^2 + \beta _\alpha (\rho )^2 (1 - \rho ^2)} = 2 \alpha + 1 - \rho ^2$ so that in particular,

$$\begin{aligned} Q_{\nu }(\rho ) = G_{\nu +1/2,\beta _{\nu +1/2}(\rho )}^{-1}(\rho ). \end{aligned}$$

As clearly $\beta _{SS}(\nu ) \le \beta _{\nu +1/2}(\rho ) \le \nu + 3/2$ for all $0 \le \rho \le 1$, we thus obtain $G_{\nu +1/2,\beta _{SS}(\nu )}^{-1}(\rho ) \le G_{\nu +1/2,\beta _{\nu +1/2}(\rho )}^{-1}(\rho ) = Q_\nu (\rho ) \le G_{\nu +1/2,\nu +3/2}^{-1}(\rho )$.

Writing $\Delta (\sigma ) = \sigma + \sqrt{(\nu +2)^2 - 4(\nu +1)\sigma } - (\nu + 2)$ we have $G_{\nu ,\nu +2}^{-1}(\rho ) - Q_\nu (\rho ) = \Delta (\rho ^2) \rho / (1 - \rho ^2)$. As $\Delta '(\sigma ) = 1 - 2(\nu +1) / \sqrt{(\nu +2)^2 - 4(\nu +1)\sigma }$ and $\Delta ''(\sigma ) = - 4 (\nu +1)^2 ((\nu +2)^2 - 4(\nu +1)\sigma )^{-3/2}$, $\Delta$ is strictly concave with its unique maximum at the solution $\sigma ^*$ of $\Delta '(\sigma ) = 0$, or equivalently $(\nu +2)^2 - 4(\nu +1)\sigma = 4(\nu +1)^2$, from which

$$\begin{aligned} \sigma ^* = \frac{(\nu +2)^2 - 4(\nu +1)^2}{4(\nu +1)} = \frac{-3\nu ^2 - 4\nu }{4(\nu +1)}, \end{aligned}$$

which is non-positive for $\nu \ge 0$. Thus, $\Delta$ is decreasing on $[0, 1]$. As $\Delta (0) = 0$, we obtain that for $0 < \rho < 1$, $\Delta (\rho ^2) < 0$ and hence $G_{\nu ,\nu +2}^{-1}(\rho ) < Q_\nu (\rho )$.

### **Corollary 2**

Let $\nu \ge 0$ and $0 \le \rho < 1$. Then

$$\begin{aligned} \left| R_{\nu }^{-1}(\rho ) - Q_\nu (\rho ) \right| \le \rho g(\nu ). \end{aligned}$$

### *Proof*

Straightforward from combining Theorems 1, 2 and 3.

We thus see that the Dhillon–Sra approximation is not invalidated by the available inverse Amos-type bounds (in the sense of being outside the range provided by these bounds), and has the same maximal approximation error as these bounds (indicating that it is indeed a good approximation).

### **Theorem 4**

Let $\nu \ge 0$. Then as $\rho \rightarrow 1-$,

$$\begin{aligned} R_{\nu }^{-1}(\rho ) - G_{\nu +1/2,\beta _{SS}(\nu )}^{-1}(\rho ) = O(\rho - 1) \end{aligned}$$

and

$$\begin{aligned} R_{\nu }^{-1}(\rho ) - Q_\nu (\rho ) = - \frac{1}{2} + O(\rho - 1). \end{aligned}$$

### *Proof*

Using the asymptotic expansion of $I_\nu$ for large argument (e.g.,Watson 1995, Formula 7.23.2), one can show that for arbitrary $\nu$,

6 $$\begin{aligned} R_\nu (t) = 1 - \frac{\nu + 1/2}{t} + \frac{\nu ^2 - 1/4}{2t^2} + O(1/t^3), \qquad t \rightarrow \infty , \end{aligned}$$

see also (Schou (1978), Eq. 6, assuming $\nu \ge 0$). Thus, we have $R_{\nu }^{-1}(\rho ) = \omega _{-1} / (\rho - 1) + \omega _0 + O(\rho - 1)$ as $\rho \rightarrow 1-$ with $\omega _{-1} \ne 0$, and the coefficients of this approximation can be determined by rewriting $\rho = R_\nu (t) \approx 1 + \alpha _1 / t + \alpha _2 / t^2$ as $(\rho -1) t^2 - \alpha _1 t - \alpha _2 \approx 0$ to obtain

$$\begin{aligned} t = R_\nu ^{-1}(\rho ) \approx \frac{\alpha _1 + \sqrt{\alpha _1^2 + 4 \alpha _2 (\rho - 1)}}{2(\rho -1)} = \frac{\alpha _1}{\rho -1} + \frac{\alpha _2}{\alpha _1} + O(\rho -1), \end{aligned}$$

giving (with $\alpha _1 = - (\nu + 1/2)$ and $\alpha _2 = (\nu -1/2)(\nu +1/2) / 2$)

$$\begin{aligned} R_\nu ^{-1}(\rho ) = - \frac{\nu +1/2}{\rho - 1} - \frac{\nu -1/2}{2} + O(\rho - 1), \qquad \rho \rightarrow {1-}. \end{aligned}$$

For $$\rho \rightarrow 1-$$,

$$\begin{aligned} \frac{\rho }{1 - \rho ^2}&= - \frac{(\rho - 1) + 1}{(\rho - 1) (2 + (\rho - 1))} \\&= - \frac{1}{2} \left( \frac{1}{\rho -1} + 1 \right) \left( 1 - \frac{\rho - 1}{2} + \frac{(\rho - 1)^2}{4} + O((\rho -1)^3) \right) \\&= - \frac{1}{2} \left( \frac{1}{\rho -1} + \frac{1}{2} - \frac{\rho - 1}{4} + O((\rho -1)^2) \right) . \end{aligned}$$

Hence, if $f(\rho ) = \delta _0 + \delta _1 (\rho - 1) + O((\rho - 1)^2)$ as $\rho \rightarrow 1$,

$$\begin{aligned} \frac{\rho }{1 - \rho ^2} f(\rho )&= - \frac{\delta _0}{2} \frac{1}{\rho - 1} - \frac{\delta _0 + 2 \delta _1}{4} + O(\rho - 1) \end{aligned}$$

as $\rho \rightarrow 1-$. In particular, as for $\alpha > 0$

$$\begin{aligned} \sqrt{\rho ^2 \alpha ^2 + (1 - \rho ^2) \beta ^2}&= \sqrt{\alpha ^2 + (\beta ^2 - \alpha ^2) (1 - \rho ^2)} \\&= \alpha \left( 1 + \frac{\beta ^2 - \alpha ^2}{2\alpha ^2} (1 - \rho ^2) + O((\rho - 1)^2) \right) \\&= \alpha - \frac{\beta ^2 - \alpha ^2}{\alpha } (\rho - 1) + O((\rho - 1)^2) \end{aligned}$$

as $$\rho \rightarrow 1$$,

$$\begin{aligned} G_{\alpha ,\beta }^{-1}(\rho )&= \frac{\rho }{1 - \rho ^2} \left( 2\alpha - \frac{\beta ^2 - \alpha ^2}{\alpha } (\rho - 1) + O((\rho - 1)^2) \right) \\&= - \frac{\alpha }{\rho - 1} - \frac{1}{4}\left( 2\alpha - 2\frac{\beta ^2 - \alpha ^2}{2\alpha }\right) + O(\rho - 1) \\&= - \frac{\alpha }{\rho - 1} - \alpha + \frac{\beta ^2}{2\alpha } + O(\rho - 1), \qquad \rho \rightarrow {1-}. \end{aligned}$$

For $\alpha = \nu + 1/2$ and $\beta = \beta _{SS}(\nu )$ we have $- \alpha + \beta ^2 / (2\alpha ) = -\nu /2 + 1/4$ so that

$$\begin{aligned} G_{\nu +1/2,\beta _{SS}(\nu )}^{-1}(\rho ) = - \frac{\nu +1/2}{\rho - 1} - \frac{\nu - 1/2}{2} + O(\rho - 1) \end{aligned}$$

and hence $R_\nu ^{-1}(\rho ) - G_{\nu +1/2,\beta _{SS}(\nu )}^{-1}(\rho ) = O(\rho - 1)$ as $\rho \rightarrow 1-$.

As $$2(\nu +1) - \rho ^2 = (2\nu + 1) - 2(\rho - 1) - (\rho - 1)^2$$,

$$\begin{aligned} Q_\nu (\rho ) = - \frac{\nu +1/2}{\rho - 1} - \frac{2\nu - 3}{4} + O(\rho -1), \qquad \rho \rightarrow {1-}. \end{aligned}$$

As

$$\begin{aligned} - \frac{\nu - 1/2}{2} - \left( -\frac{2\nu - 3}{4}\right) = -\frac{1}{2}, \end{aligned}$$

we thus have $R_\nu ^{-1}(\rho ) - Q_\nu (\rho ) = -1/2 + O(\rho - 1)$ as $\rho \rightarrow 1-$, and the proof is complete.

## Nåsell bounds

Nåsell (1978) gives families $L_{\nu ,k,m}$ and $U_{\nu ,k,m}$ of *rational* lower and upper bounds for $R_\nu$, which converge to $R_\nu$ as $m \rightarrow \infty$ or $k \rightarrow \infty$ (Nåsell 1978, Theorems 2 and 3). These bounds can be used for obtaining bounds for $R_\nu ^{-1}$ by applying numerical root finding techniques either directly to equations of the form $P(t) / Q(t) = \rho$ with polynomials $P$ and $Q$, or by rewriting the equations of this form as $R(t) = P(t) - \rho Q(t) = 0$ and then determining a suitable root of the polynomial $R$. “Simple” closed form expressions can be obtained when root finding amounts to solving a quadratic equation.

As $R_\nu (t)$ tends to 0 and 1 for $t \rightarrow 0$ and $\infty$, respectively, we thus restrict ourselves to Nåsell bounds exhibiting the same limits, and having numerator and denominator degrees at most 2. This leaves (Nåsell 1978, Appendix) the lower bounds $L_{\nu ,1,0} < L_{\nu ,2,0}$ and the upper bounds $U_{\nu ,1,1}$ and $U_{\nu ,3,0} < U_{\nu ,2,0}$, where the inequalities again follow from Theorems 2 and 3 in the reference. Neither of $U_{\nu ,1,1}$ and $U_{\nu ,3,0}$ dominates the other, as they have different orders of approximation at 0 and $\infty$. In fact, writing

$$\begin{aligned} U_{\nu ,1,1}(t) = t \frac{P_{\nu ,1,1}(t)}{Q_{\nu ,1,1}(t)} = t \frac{2(\nu +2) + t}{4(\nu +1)(\nu +2) + 2(\nu +1)t + t^2} \end{aligned}$$

and

$$\begin{aligned} U_{\nu ,3,0}(t) = t \frac{P_{\nu ,3,0}(t)}{Q_{\nu ,3,0}(t)} = t \frac{\frac{1}{2}(\nu + 1/2) + t}{ (\nu +1/2)(\nu +1) + \frac{3}{2}(\nu + 1/2) t + t^2}, \end{aligned}$$

it is readily verified that

$$\begin{aligned} U_{\nu ,1,1}(t) - U_{\nu ,3,0}(t) = \frac{(\nu + 5/2) t^2 (t - (\nu +2))}{Q_{\nu ,1,1}(t) Q_{\nu ,3,0}(t)}. \end{aligned}$$

This implies that with $t_\nu = \nu +2$, $U_{\nu ,1,1}(t) < U_{\nu ,3,0}(t)$ for $0 < t < t_\nu$, and $U_{\nu ,1,1}(t) > U_{\nu ,3,0}(t)$ for $t > t_\nu$.

The best lower bound $L_{\nu ,2,0}$ which only involves solving a quadratic equation is given by

$$\begin{aligned} L_{\nu ,2,0} = t\frac{(\nu + 3/2) + t}{2(\nu + 1)(\nu + 3/2) + 2(\nu + 1)t + t^2}. \end{aligned}$$

### **Theorem 5**

Let $\nu \ge 0$. Then for all $t > 0$,

$$\begin{aligned} L_{\nu ,2,0}(t) < G_{\nu +1/2, \nu +3/2}(t) \end{aligned}$$

and

$$\begin{aligned} \min \left( G_{\nu ,\nu +2}(t), G_{\nu +1/2,\beta _{SS}(\nu )}(t)\right) < \min \left( U_{\nu ,1,1}(t), U_{\nu ,3,0}(t)\right) . \end{aligned}$$

### *Proof*

All Nåsell bounds under consideration are of the form

$$\begin{aligned} t \frac{P(t)}{Q(t)}, \qquad P(t) = t + \gamma , \quad Q(t) = t^2 + \delta _1 t + \delta _0 \end{aligned}$$

with non-negative coefficients $\gamma$, $\delta _1$ and $\delta _0$. All Amos-type bounds $G_{\alpha , \beta }$ under consideration have $\alpha \le \beta$.

To show that the Amos-type bounds dominate these Nåsell bounds we have to investigate when

$$\begin{aligned} \frac{P(t)}{Q(t)} - \frac{1}{\alpha + \sqrt{t^2 + \beta ^2}} \end{aligned}$$

has no zeros on $(0, \infty )$, or equivalently, when

$$\begin{aligned} \Delta (t) = \left( Q(t) - \alpha P(t)\right) ^2 - \left( t^2 + \beta ^2\right) P(t)^2 \end{aligned}$$

has no zeros on $(0, \infty )$. Note that

$$\begin{aligned} \Delta (t) = \left( Q(t) - \left( \alpha + \sqrt{t^2 + \beta ^2}\right) P(t) \right) \left( Q(t) - \left( \alpha - \sqrt{t^2 + \beta ^2}\right) P(t) \right) , \end{aligned}$$

where the second term is always positive for $t > 0$ and $\alpha \le \beta$. Hence, $\Delta (t) > 0$ for all $t > 0$ is equivalent to $Q(t) - (\alpha + \sqrt{t^2 + \beta ^2}) P(t) > 0$, or $P(t) / Q(t) < 1 / (\alpha + \sqrt{t^2 + \beta ^2})$ for all $t > 0$; $\Delta (t) < 0$ for all $t > 0$ is equivalent to $P(t) / Q(t) > 1 / (\alpha + \sqrt{t^2 + \beta ^2})$ for all $t > 0$.

Writing

$$\begin{aligned} Q(t) - \alpha P(t) = t^2 + (\delta _1 - \alpha ) t + (\delta _0 - \alpha \gamma ) = t^2 + \omega _1 t + \omega _0, \end{aligned}$$

we obtain

$$\begin{aligned} \Delta (t)&= (t^2 + \omega _1 t + \omega _0)^2 - (t^2 + \beta ^2) (t + \gamma )^2 \\&= 2 (\omega _1 - \gamma ) t^3 + (\omega _1^2 + 2 \omega _0 - \beta ^2 - \gamma ^2) t^2 \!+\! 2 (\omega _1 \omega _0 \!-\! \beta ^2 \gamma ) t + (\omega _0^2 - \beta ^2 \gamma ^2). \end{aligned}$$

Comparing $L_{\nu ,2,0}$ to $G_{\nu +1/2,\nu +3/2}$, we have $\gamma = \nu + 3/2$, $\delta _0 = 2 (\nu +1) (\nu + 3/2)$ and $\delta _1 = 2 (\nu +1)$, from which $\omega _0 = \delta _0 - \alpha \gamma = 2 (\nu + 1) (\nu +3/2) - (\nu + 1/2) (\nu + 3/2) = (\nu + 3/2)^2 = \beta ^2$ and $\omega _1 = \delta _1 - \alpha = \nu + 3/2 = \beta$, giving

$$\begin{aligned} 2 (\omega _1 - \gamma )&= 0, \\ \omega _1^2 + 2 \omega _0 - \beta ^2 - \gamma ^2&= \beta ^2, \\ 2 (\omega _1 \omega _0 - \beta ^2 \gamma )&= 0, \\ \omega _0^2 - \beta ^2 \gamma ^2&= 0 \end{aligned}$$

so that $\Delta (t) = \beta ^2 t^2 > 0$ for all $t > 0$. Hence, we have $L_{\nu ,1,0}(t) < L_{\nu ,2,0}(t) < G_{\nu +1/2, \nu +3/2}(t)$ for all $t > 0$.

Comparing $U_{\nu ,1,1}$ to $G_{\nu ,\nu +2}$, we have $\gamma = 2(\nu +2) = 2 \beta$, $\delta _0 = 4(\nu +1)(\nu +2)$ and $\delta _1 = 2(\nu +1)$, from which $\omega _0 = \delta _0 - \alpha \gamma = 4 (\nu +1) (\nu + 2) - 2 \nu (\nu + 2) = 2 (\nu + 2)^2 = 2 \beta ^2$ and $\omega _1 = \delta _1 - \alpha = 2 (\nu + 1) - \nu = \nu + 2 = \beta$, giving

$$\begin{aligned} 2 (\omega _1 - \gamma )&= -2 \beta , \\ \omega _1^2 + 2 \omega _0 - \beta ^2 - \gamma ^2&= 0, \\ 2 (\omega _1 \omega _0 - \beta ^2 \gamma )&= 0, \\ \omega _0^2 - \beta ^2 \gamma ^2&= 0, \end{aligned}$$

so that $\Delta (t) = - 2 \beta t^3 < 0$ for all $t > 0$. Hence, $G_{\nu ,\nu +2}(t) < U_{\nu ,1,1}(t)$ for all $t > 0$.

Finally, comparing $U_{\nu ,3,0}$ to $G_{\nu +1/2,\beta _{SS}(\nu )}$, we have $\gamma = \frac{1}{2} (\nu + 1/2) = \alpha / 2$, $\delta _0 = (\nu +1/2) (\nu + 1) = \alpha (\alpha + 1/2)$. $\delta _1 = \frac{3}{2} (\nu + 1/2) = 3\alpha /2$, from which $\omega _0 = \delta _0 - \alpha \gamma = \alpha (\alpha + 1/2) - \alpha ^2 / 2 = \alpha (\alpha + 1) / 2 = \beta ^2 / 2$ and $\omega _1 = \delta _1 - \alpha = 3\alpha /2 - \alpha = \alpha /2$, giving

$$\begin{aligned} 2 (\omega _1 - \gamma )&= 0, \\ \omega _1^2 + 2 \omega _0 - \beta ^2 - \gamma ^2&= 0, \\ 2 (\omega _1 \omega _0 - \beta ^2 \gamma )&= - \alpha \beta ^2 / 2, \\ \omega _0^2 - \beta ^2 \gamma ^2&= \beta ^2 (\beta ^2 - \alpha ^2) / 4, \end{aligned}$$

so that with $\beta ^2 - \alpha ^2 = (\nu +1/2)(\nu +3/2) - (\nu +1/2)^2 = \nu + 1/2 = \alpha$ we get

$$\begin{aligned} \Delta (t) = - \frac{\alpha \beta ^2}{2} t + \frac{\beta ^2\alpha }{4} = \frac{\beta ^2\alpha }{4} (1 - 2t), \end{aligned}$$

which is negative for $t > 1/2$, so that $G_{\nu +1/2,\beta _{SS}(\nu )}(t) < U_{\nu ,3,0}(t)$ for all $t > 1/2$.

As $U_{\nu ,1,1}(t) < U_{\nu ,3,0}(t)$ for $0 < t < t_\nu = \nu + 2$, we infer that

$$\begin{aligned} \min \left( G_{\nu ,\nu +2}(t), G_{\nu +1/2,\beta _{SS}(\nu )}(t)\right) < \min \left( U_{\nu ,1,1}(t), U_{\nu ,3,0}(t)\right) \end{aligned}$$

for all $t > 0$, and the proof is complete.

As the Nåsell bounds considered in the previous theorem are dominated by the Amos-type bounds used for $R_\nu$, the same must be true for the respective inverses.

### **Corollary 3**

Let $\nu \ge 0$ and $0 \le \rho < 1$. Then

$$\begin{aligned} R_\nu ^{-1}(\rho ) \le G_{\nu +1/2,\nu +3/2}^{-1}(\rho ) < L_{\nu ,2,0}^{-1}(\rho ) \end{aligned}$$

and

$$\begin{aligned} \max \left( U_{\nu ,1,1}^{-1}(\rho ), U_{\nu ,3,0}^{-1}(\rho ) \right) \le \max \left( G_{\nu ,\nu +2}^{-1}(\rho ), G_{\nu +1/2, \beta _{SS}(\nu )}^{-1}(\rho ) \right) \le R_{\nu }^{-1}(\rho ). \end{aligned}$$

### *Proof*

Straightforward from the previous theorem and Theorem 1.

## Numerical comparisons

In the following we compare the number of iterations required to reach convergence by two different algorithms based on nested intervals for finding roots using (1) the Tanabe et al. (2007) and (2) the newly established bounds for initialization. The two algorithms used are (a) a one-dimensional root finding algorithm as implemented in function uniroot() available in R (R Core Team 2013) and (b) the variant of the Newton-Fourier method for the case of strictly increasing concave functions (see e.g., Atkinson 1989, pp. 62–64). Concavity of $R_{\nu }$ can be established by using that $R_{\nu }$ is the pointwise minimum of a set of Amos-type functions (see Hornik and Grün 2013, Theorem 11). It is straightforward to show that the second derivative of these Amos-type functions is non-positive and hence $R_{\nu }$ is concave, because it is the pointwise minimum of concave functions.

The numbers of iterations required are determined for a range of different $d$ and $\kappa$ values which are selected similar to those used in Sra (2012), i.e., $\kappa$ $=$ 100, 500, 1,000, 5,000, 10,000, 20,000, 50,000, 10,000 and $d$ $=$ 500, 1,000, 5,000, 10,000, 20,000, 10,000. Each value of $d$ is combined with each value of $\kappa$ and they are used to determine the corresponding $\rho$. For the given $d$ and implied $\rho$ the root finding algorithms are employed to determine $\kappa$ again. For uniroot() convergence is assessed using argument tol and the Newton-Fourier algorithm is stopped if the relative distance of the midpoint to the endpoints of the interval is smaller than tol. This pre-specified precision value tol is varied and set to values $10^{e}$ for exponents $e \in \{-6$, $-8$, $-10$, $-$12}.

The results are given in Table 1. For the Newton-Fourier algorithm the newly established bounds always require the same or a smaller number of iterations. In 31 % of the cases one iteration less is required, reducing the number of iterations required by 60 or 20 %. When using uniroot(), there is an improvement achievement in 55 % of the cases, the same number of iterations are required in 42 % of the cases and only in 3 % of the cases a deterioration occurs and one iteration more is required when using the newly established bounds for initialization.

**Table 1:** Number of iterations required by uniroot() (top) and by the Newton-Fourier algorithm (bottom) using the Tanabe et al. (2007) bounds (T07) and the newly established bounds (New) for initialization

T07	New					
	0	1	2	3	4	5
1	4	3	0	0	0	0
2	2	22	32	2	0	0
3	0	5	46	36	3	0
4	0	0	5	14	9	0
5	0	0	4	3	1	1

T07	New					
	1	2				
1	87	0				
2	52	45				
3	0	8				

Overall it has to be noted that the number of iterations is rather small to reach convergence when either of the two sets of intervals is used for initialization. However, the general tendency to require even less iterations of the newly established bounds will nevertheless be of interest from a computational point of view if these roots are solved repeatedly, which is required when for example the expectation-maximization algorithm is used to estimate mixtures of von Mises–Fisher distributions (Banerjee et al. 2005).

## References

[CR1] Amos DE (1974) Computation of modified Bessel functions and their ratios. Math Comput 28(125):239–251. http://www.jstor.org/stable/2005830

[CR2] Atkinson KE (1989). An introduction to numerical analysis.

[CR3] Banerjee A, Dhillon IS, Ghosh J, Sra S (2005) Clustering on the unit hypersphere using von Mises–Fisher distributions. J Mach Learn Res 6:1345–1382. http://jmlr.csail.mit.edu/papers/v6/banerjee05a.html

[CR4] Dhillon IS, Sra S (2003) Modeling data using directional distributions. Tech. Rep. TR-03-06, Department of Computer Sciences, The University of Texas at Austin. ftp://ftp.cs.utexas.edu/pub/techreports/tr03-06.ps.gz

[CR5] Gautschi W, Slavik J (1978) On the computation of modified Bessel function ratios. Math Comput 32(143):865–875. http://www.jstor.org/stable/2006491

[CR6] Hornik K, Grün B Amos-type bounds for modified Bessel function ratios. J Math Anal Appl. doi:10.1016/j.jmaa.2013.05.070 10.1016/j.jmaa.2013.05.070 PMC4047631 24926105

[CR7] Mardia KV, Jupp PE (1999) Directional statistics. Probability and Statistics. Chichester, UK

[CR8] Nåsell I (1978). Rational bounds for ratios of modified Bessel functions. SIAM J Math Anal.

[CR9] R Core Team (2013) R: a language and environment for statistical computing. R Foundation for Statistical Computing, Vienna. http://www.R-project.org/

[CR10] Schou G (1978). Estimation of the concentration parameter in von Mises-Fisher distributions. Biometrika.

[CR11] Simpson HC, Spector SJ (1984). Some monotonicity results for ratios of modified Bessel functions. Quart Appl Math.

[CR12] Song H, Liu J, Wang G (2012). High-order parameter approximation for von Mises-Fisher distributions. Appl Math Comput.

[CR13] Sra S (2012). A short note on parameter approximation for von Mises-Fisher distributions: and a fast implementation of $$I_s(x)$$. Comput Stat.

[CR14] Tanabe A, Fukumizu K, Oba S, Takenouchi T, Ishii S (2007). Parameter estimation for von Mises-Fisher distributions. Comput Stat.

[CR15] Tretter MJ, Walster GW (1980) Further comments on the computation of modified Bessel function ratios. Math Comput 35(151):937–939. http://www.jstor.org/stable/2006205

[CR16] Watson GN (1995) A treatise on the theory of bessel functions, 2nd edn. Cambridge mathematical library, Cambridge University Press. doi:10.2277/0521483913; http://www.cambridge.org/9780521483919

[CR17] Yuan L, Kalbfleisch JD (2000). On the Bessel distribution and related problems. Ann Inst Stat Math.

